# Investigation of the pathogen-specific antibody response in periprosthetic joint infection

**DOI:** 10.1007/s15010-024-02285-y

**Published:** 2024-05-31

**Authors:** Viktor Janz, Anastasia Rakow, Leonie Schröder, André Hofer, Sergej Wiebe, Janosch Schoon, Stefan Weiss, Barbara M. Bröker, Georgi I. Wassilew, Dina Raafat

**Affiliations:** 1 Center for Orthopaedics, Trauma Surgery and Rehabilitation Medicine, University Medicine Greifswald, 17475 Greifswald, Germany; 2 Sporthopaedicum, 93053 Regensburg, Germany; 3 Institute of Immunology, University Medicine Greifswald, 17475 Greifswald, Germany; 4 Interfaculty Institute for Genetics and Functional Genomics, University Medicine Greifswald, 17475 Greifswald, Germany; 5 Department of Microbiology and Immunology, Faculty of Pharmacy, Alexandria University, 21521 Alexandria, Egypt

**Keywords:** Periprosthetic joint infection, PJI, Pathogen-specific antibody response, Serology, Disease monitoring, Infection Array

## Abstract

**Purpose:**

Periprosthetic joint infections (PJIs) are a very demanding complication of arthroplasty. Diagnosis of PJI and pathogen identification pose considerable challenges in clinical practice. We hypothesized that the pathogen-specific immune response to PJI reflects the infection process, provides clinically relevant information on disease course, and has the potential to further optimize antimicrobial therapy.

**Methods:**

We conducted a prospective matched cohort pilot study with 13 patients undergoing two-stage septic revision arthroplasty (PJI patients) between 06/2020 and 06/2021, as well as 11 control patients undergoing one-stage aseptic revision arthroplasty (Non-PJI patients). Pre-, intra- and postoperative serum samples were collected at standardized time points. We developed a custom Luminex®-based quantitative bead-based suspension array (Infection Array; IA), and used it for simultaneous measurement of antibody specificities against 32 pathogens commonly associated with PJI in 267 serum samples.

**Results:**

The IA was able to trace the dynamics of the pathogen-specific humoral immune response in all patients against PJI-related pathogens, prominently coagulase-negative staphylococci and streptococci. Pathogen-specific serum antibody titers declined in 62% of PJI patients over the course of treatment, while no changes in antibody titers were observed in 82% of Non-PJI patients during this study. Our serological data strongly suggested that antibody signatures reflect an immune response to microbial invasion.

**Conclusion:**

Our results provide insights into the pathophysiology of PJI and information on the individual disease courses. The IA is therefore a promising and novel serological tool of high resolution for monitoring the immunoproteomic footprints of infectious pathogens in the course of PJI.

**Supplementary Information:**

The online version contains supplementary material available at 10.1007/s15010-024-02285-y.

## Introduction

Periprosthetic joint infections (PJIs) remain a very challenging complication of total hip and total knee arthroplasties (THA and TKA, respectively). PJI is one of the three most common reasons for revision of THA and TKA, placing a substantial financial burden on healthcare systems worldwide [1–6].

Due to the large number of affected patients and the high clinical and financial impact of PJI, its treatment has evolved significantly over the last decades, and should be performed by highly specialized interdisciplinary teams of infectious disease specialists, orthopaedic surgeons and microbiologists [7]. Despite this high specialization, the treatment of PJIs remains centered upon pathogen identification via microbiological culture, surgical revision and antimicrobial therapy. In 20–50% of PJIs, the causative microbial species cannot be identified by routine microbiological cultures [8–12]. These cases are referred to as “culture-negative PJIs”, which consequently entail an empiric antimicrobial therapy [13–17].

Recent research developments have attempted to improve the detection of “culture-negative PJIs” through a multitude of non-culture based diagnostic methods, including molecular assays (multiplex-PCR and next-generation sequencing), serum biomarkers (interleukin-6, D-dimer), synovial fluid biomarkers (alpha-defensin, leukocyte esterase, interleukin-6, synovial-CRP), as well as multi-omic analysis [8–10, 12, 18].

Although increasingly more host-based diagnostic techniques are being introduced for immune profiling in PJI [18], to date, the patient’s individual immune response is neither considered nor monitored in the therapeutic decision-making process. We hypothesize that the pathogen-specific adaptive immune response to PJI not only reflects the infection dynamics, but also provides clinically relevant information regarding the success of treatment. To test these hypotheses, we developed a quantitative high-throughput immunoproteomic Infection Array (IA) to measure individual pathogen-specific antibody responses during PJI treatment. We used this IA in a prospective matched cohort pilot study involving patients undergoing two-stage septic revision arthroplasty due to PJI, as well as control patients undergoing one-stage aseptic revision arthroplasty for indications other than PJI.

## Materials and methods

### Study design and cohort description

**Fig. 1:**
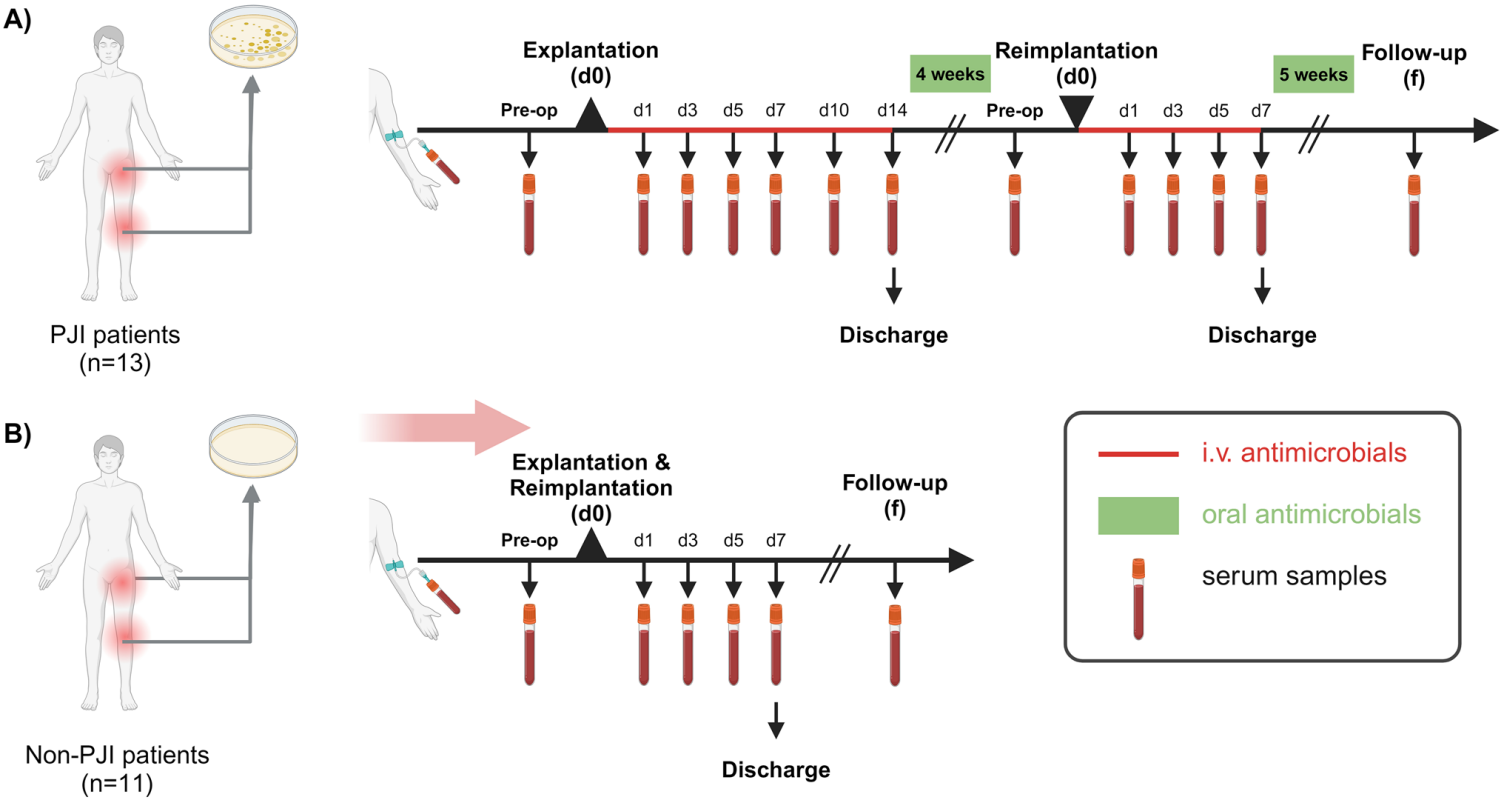
Study design of the prospective PJI pilot study, including surgical procedure, antimicrobial therapy and time points of serum sample collection. Pre-, peri- and post-operative serum samples were collected from all study participants at the indicated time points. **A** PJI patients (n = 13) undergoing two-stage septic revision arthroplasty for infected THA and TKA. Both the first-stage explanation surgery (d0) and the second-stage reimplantation surgery (d0) are followed by standardized time intervals of intravenous (red line) and oral (green box) antimicrobial therapy. **B** Non-PJI patients (n = 11) subjected to one-stage aseptic revision arthroplasty. Clinical follow-up (f) samples were collected from all study participants. The figure was created with BioRender.com

We performed a prospective monocentric matched cohort proof of concept pilot clinical study at the University Medicine Greifswald (UMG), to investigate the patients’ pathogen-specific antibody response during two-stage septic revision arthroplasty (Fig. 1). Patients undergoing one-stage aseptic revision arthroplasty were included as controls. An institutional review board approval was obtained prior to commencement of this study (Nr. BB 036/20), which was performed in accordance with the principles of the Declaration of Helsinki. Informed written consent was obtained from each of the study participants and archived according to GLP practices.

Thirteen consecutive PJI patients undergoing two-stage septic revision arthroplasty of the hip (THA, n = 11) or the knee (TKA, n = 2) between 06/2020 and 06/2021 (PJI patients) were recruited for the study (Table 1, Fig. 1A, Suppl. Table S1). PJI was diagnosed as defined by the Musculoskeletal Infection Society (MSIS), which includes multiple microbiological, histological and laboratory parameters [19].

**Table 1:** Characteristics of the study cohort including PJI (N = 13) and Non-PJI (N = 11) patients

Characteristic	PJI patients	Non-PJI patients
Number (N)	13	11
**Demographic data**		
Sex		
M (%)	12 (92%)	2 (18%)
F (%)	1 (8%)	9 (82%)
Age, mean ± SD [years]	70.9 ± 10.5	70.5 ± 10.8
**Type of total joint arthroplasty**		
Total hip arthroplasty (THA)	11	8
Total knee arthroplasty (TKA)	2	3
**Type of Revision Surgery**		
Two-staged septic exchange	13	N/A
Partial revision	N/A	5
Total revision	N/A	6
**Indication for Revision Surgery**		
Periprosthetic joint infection	13	0
Aseptic loosening	0	5
Periprosthetic fracture	0	1
Instability	0	2
Polyethylene wear / Osteolysis	0	1
Mechanical complications (Patella baja and Iliopsoas impingement)	0	2
Classification of Periprosthetic Joint Infection (PJI)*		
Early (≤ 3 months)	2	N/A
Delayed (3–24 months)	1	N/A
Late (> 24 months)	10	N/A

* PJI was classified as early, delayed or late onset according to time of infection [21, 22]

Abbreviations: N/A, not applicable

The PJI patients were matched to 11 control patients (Non-PJI patients), undergoing one-stage aseptic revision arthroplasty of the hip or the knee joint (THA, n = 8; TKA, n = 3) (Table 1, Fig. 1B, Suppl. Table S1). Matching criteria included age (± 5 years), year (± 1 year) and type of surgery. The PJI group included patients with PJI classified as early post-operative (n = 2), delayed (n = 1) and late PJI (n = 10) according to international consensus [19, 21, 22]. All of the late PJI episodes were identified to be of hematogenous origin, using previously established definition criteria [23, 24]. For clinical diagnostics and treatment, standardized microbiological and histological clinical samples (five periprosthetic tissue samples, synovial fluid samples, periprosthetic membrane) were collected intraoperatively from all participants, in accordance with current clinical standards [19, 20, 25–27].

**Fig. 2:**
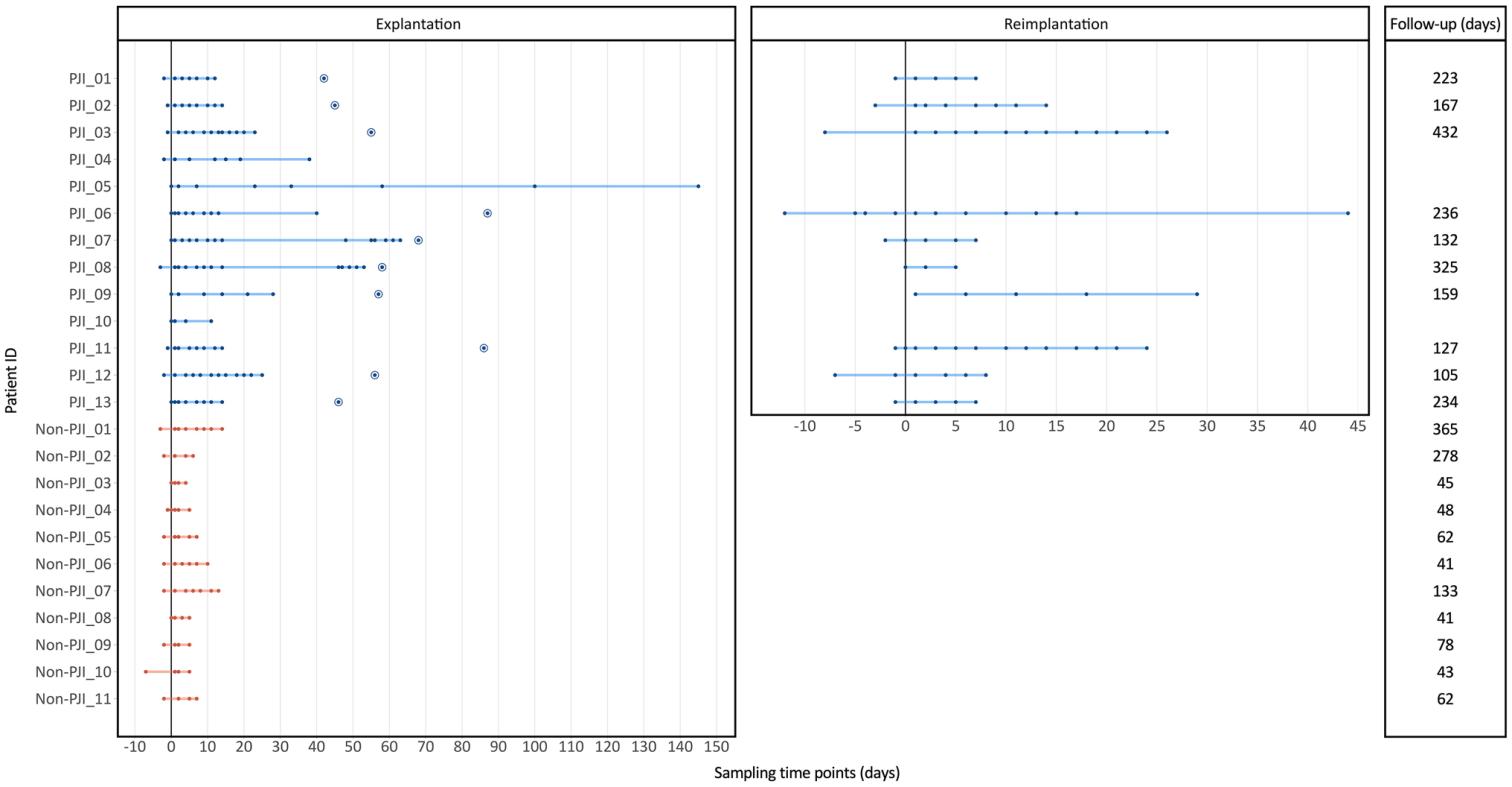
Sampling time points for the PJI pilot study. Thirteen PJI patients (blue) undergoing two-stage septic revision surgery for infected THA and TKA and 11 Non-PJI patients (red) subjected to one-stage aseptic arthroplasty revision surgery were recruited for the study over the course of one year. The figure depicts the sampling time points (in days) for each patient. Circled points in the left panel represent the day of reimplantation surgery, which is set to zero in the reimplantation plot (right panel). The follow-up samples were collected from all patients at an average of 214 days (105–432 days) and 109 days (41–365 days) post explantation surgery, for the PJI and Non-PJI groups, respectively. Three PJI patients (PJI_04, PJI_05 and PJI_10) did not undergo a reimplantation surgery. One PJI patient (PJI_09) underwent one reimplantation surgery (depicted here), but refused a second reimplantation

Serum samples for subsequent IA analysis were acquired prior to explantation surgery from the PJI patients and prior to aseptic revision surgery from the Non-PJI group (Fig. 1, Fig. 2). Additional samples were then acquired three times weekly until the patients were discharged from the hospital. In the PJI patients, this procedure was repeated before and after reimplantation surgery, i.e. implantation of a new arthroplasty implant. In addition, samples were retrieved at the final clinical follow-up visit (105–432 days and 41–365 days following explantation surgery for PJI and Non-PJI patients, respectively). Over the course of this pilot study, the pathogen-specific antibody responses were measured in a total of 267 patient samples, with an average of 15 and 6 samples for PJI and Non-PJI patients, respectively (range: 4–26 samples/patient) (Fig. 2).

### Clinical treatment of PJI

The current gold standard for PJI therapy involves using therapeutic algorithms to select for the optimal surgical treatment for PJI patients, including debridement and implant retention (DAIR), one-stage and two-stage septic arthroplasty revision surgeries [21, 28]. Two-stage septic arthroplasty revision surgery involves a standardized treatment protocol with fixed time intervals and a combination of intravenous and oral antimicrobial treatment (Fig. 1A). Treatment begins with the explantation surgery, during which the arthroplasty implant is removed and a thorough surgical debridement and lavages of the joint are performed, microbiological and histological samples are acquired as previously described [19, 20, 25–27], and an antibiotic-impregnated cement spacer may be implanted. This first-stage is typically followed by two weeks of intravenous and then four weeks of oral antimicrobial therapy [29]. If the patient’s serum C-reactive protein (CRP) is within the physiological range (<  0.5 mg/L), and local clinical signs of infection/inflammation are absent, the second surgical stage, i.e. implantation of a new arthroplasty implant, is performed. Reimplantation surgery is followed by one week of intravenous and five weeks of oral antimicrobial therapy [29]. All PJI and Non-PJI patients in our study cohort underwent routine postoperative clinical follow-ups to assess treatment success (Fig. 1, Fig. 2).

### Infection Array

**Fig. 3:**
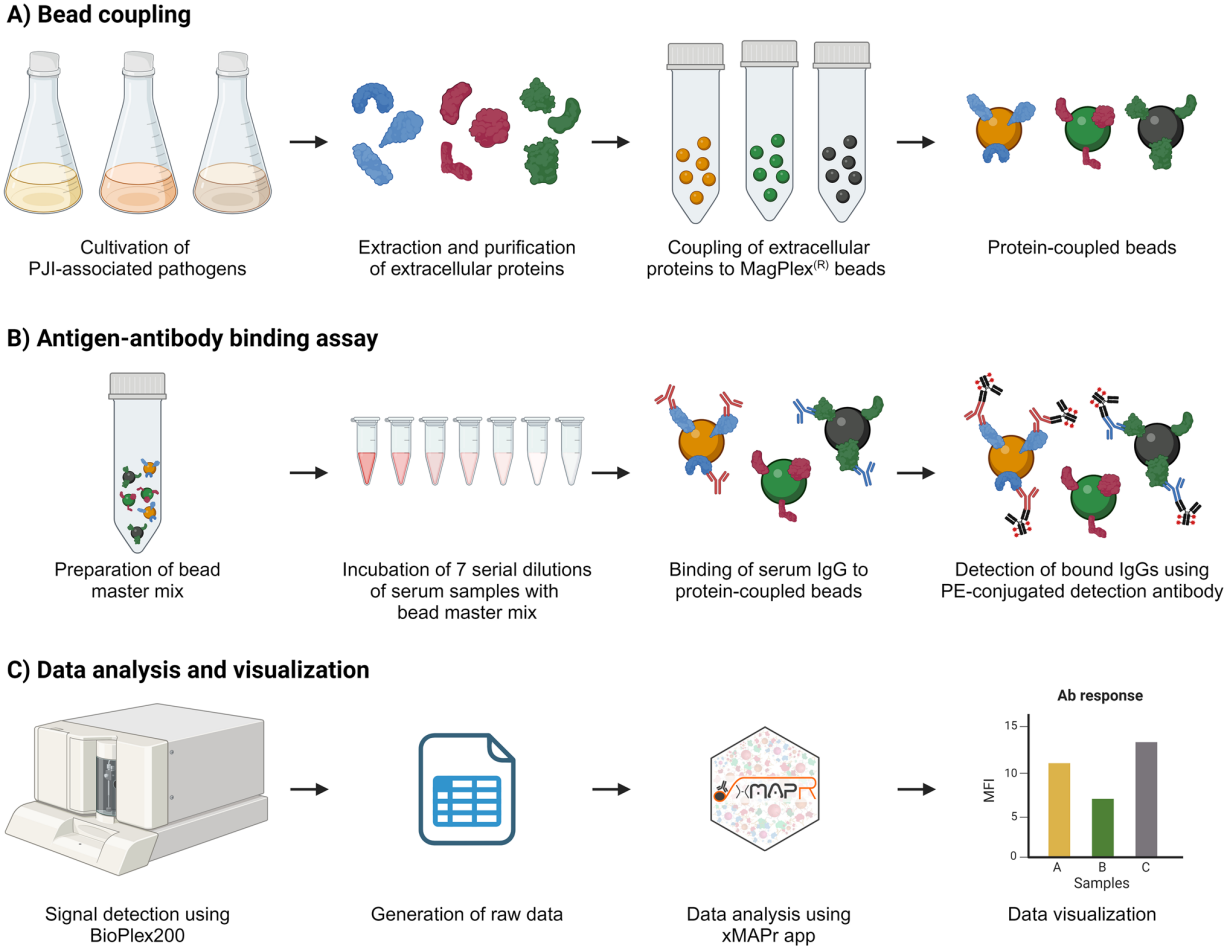
Principle and implementation of the IA. **A** Extracellular proteins were purified from 32 PJI-related pathogens and covalently coupled to magnetic MagPlex® beads, using tetanus toxoid as a control antigen. **B** Seven serial dilutions of serum samples (n = 267) were prepared and incubated with the protein-coupled bead master mix. Bound serum antibodies were detected using a PE-conjugated detection antibody. **C** Antibody binding was determined on a BioPlex® 200 system, and was quantified using the xMAPr app. Data analysis and visualization were done with GraphPad Prism (v8.0.1). The figure was created with BioRender.com

The Infection Array (IA) is an in-house developed bead-based suspension array (Luminex^®^-based) for multiplex measurement of antibody specificities directed against 32 pathogens corresponding to 30 different species that are commonly associated with PJI (Fig. 3). The IA was prepared by cultivating the 32 pathogens and purifying their extracellular proteins. These 32 extracellular protein extracts were adjusted to a concentration of 10 mg/mL and then covalently coupled to magnetic microspheres (MagPlex^®^ microspheres, beads) with unique spectral signatures. Tetanus toxoid (TT) served as a control antigen in a 33-plex approach (Table 2). All samples were blinded with respect to the results of microbiological culture before processing. Antibody binding was detected on a BioPlex^®^ 200 system and quantified using a dedicated app (xMAPr) [30].

**Table 2:** The 33-plex Infection Array (IA)-panel

Nr	Species / Protein	Abbreviation	Cut-off values for IA^b^
1	*Corynebacterium striatum*	C. stri	2.17
2	*Cutibacterium acnes*	C. acn	2.33
3	*Clostridioides difficile*	C. diff	2.63
4	*Staphylococcus aureus*	S. au1	2.84
5	*Staphylococcus aureus*	S. au2	2.02
6	*Staphylococcus epidermidis*	S. epi	2.11
7	*Staphylococcus haemolyticus*	S. hae	2.48
8	*Staphylococcus hominis*	S. hom	2.18
9	*Staphylococcus lugdunsensis*	S. lug	3.02
10	*Staphylococcus warneri*	S. war	3.25
11	*Enterococcus faecalis*	E. fcalis	2.43
12	*Enterococcus faecium*	E. fcium	3.19
13	*Streptococcus pneumoniae*	S. pneu	2.34
14	*Streptococcus mitis*	S. mitis	2.30
15	*Streptococcus oralis*	S. oral	1.97
16	*Streptococcus sanguinis*	S. san	3.06
17	*Streptococcus gallolyticus*	S. gal	3.36
18	*Haemophilus influenzae*	H. infl	2.21
19	*Klebsiella aerogenes*	K. aero	2.27
20	*Klebsiella pneumoniae*	K. pneu	2.45
21	*Klebsiella oxytoca*	K. oxy	2.66
22	*Enterobacter cloacae*	E. clo	2.22
23	*Escherichia coli*	E. col	2.36
24	*Proteus mirabilis*	P. mir	2.21
25	*Serratia marcescens*	S. marc	1.89
26	*Pseudomonas aeruginosa*	P. aeru1	3.62
27	*Pseudomonas aeruginosa*	P. aeru2	2.68
28	*Acinetobacter baumannii*	A. baum	2.43
29	*Stenotrophomonas maltophilia*	S. malt	2.62
30	*Moraxella catarrhalis*	M. cat	1.97
31	*Legionella pneumophila*	L. pneu	2.23
32	*Candida albicans*	C. albi	2.77
33	Tetanus Toxoid^a^	TT	1.91

^a^ Tetanus Toxoid (TT; Statens Serum Institute, Denmark) was included as a control antigen

^b^ Pathogen-specific cut-off values for IA were calculated by using data from all 24 patients included in the study. After removing outliers using the ROUT method (Q = 0.1%), the remaining data were used to calculate ratios (ratio (dx/df)), and the pathogen-specific cut-off value was set at mean of ratio (dx/df) + 5 × SD

#### Bacterial extracellular protein precipitation and purification

Extracellular proteins (ECPs) from 32 different pathogens were prepared as previously described [31]. A list of all pathogens used in the 33-plex IA-panel, together with their respective abbreviations and culture conditions, is provided in Suppl. Table S2. Briefly, the pathogens were cultivated under optimal growth conditions until the early stationary growth phase, and the culture supernatants were collected by centrifugation at 8000 × *g* for 10 min at 4 °C and filtered through a 0.22-μm membrane to remove residual bacteria. The ECPs in the supernatants were precipitated using a pre-chilled 100% trichloroacetic acid solution, followed by incubation for 48 h at 4 °C. Proteins were collected (8000 × *g*, 1 h, 4 °C) and then resuspended in 70% ethanol. After several washing steps in 70 and 96% ethanol, the pellets were dried in a SpeedVac (V-AL; Break: ON; Temp: 30 °C). Finally, the precipitated proteins were rehydrated in 5% SDS. The protein concentration was determined using the Pierce™ BCA protein assay kit (Thermo Scientific, USA), and the extracts were stored at −80 °C.

#### Coupling of ECPs to magnetic microspheres

Each protein extract was covalently coupled to MagPlex^®^ magnetic microspheres (Luminex Corp., USA; beads) of unique color-codes using a final concentration of 0.5 µg protein/1.25 × 10^4^ beads (for TT: 0.25 µg protein/1.25 × 10^4^ beads). The coupling was carried out at RT following a standard carbodiimide coupling procedure as described before [30, 32]. The individual antigen-coupled bead populations were combined into a bead master mix that constitutes the 33-plex IA. The aliquoted master mix was stored at 4 °C.

#### Antigen/antibody binding assay

To accurately determine IgG titers against the different pathogens included in the IA, seven-step dilution series (1:20–1:312,500 dilutions) were prepared from the serum samples. A human reference serum (Cat. # HMSRM, Bioreclamation IVT, USA) was included on each plate for subsequent normalization of data. After thoroughly resuspending the bead master mix, it was added to the wells of half-well 96-well plates (Greiner Bio-One GmbH, Germany) at a final count of 1,250 beads/50 µL/well. The bead storage solution was removed, and the diluted samples/reference serum were added to the beads (50 µL/well). The plates were sealed with aluminum foil and incubated in the dark on a shaker (4 °C, 18 h, 900 rpm) to allow for binding of serum antibodies to the protein-coupled beads. Unbound antibodies were removed by washing, and bound IgG antibodies were detected by incubation (in the dark, 900 rpm, 90 min, RT) with an RPE-conjugated detection antibody (R-PE AffiniPure F(ab’)_2_ Fragment Goat Anti-Human IgG, Fcγ fragment specific; Cat. No.: 109-116-098; Jackson ImmunoResearch; USA), diluted in bead buffer to a final concentration of 5 µg/mL.

After three additional washing steps to remove unbound detection antibodies, beads were resuspended in sheath fluid (xMap Sheath Fluid, Luminex Corp., USA) in the dark on a plate shaker (900 rpm, at least 1 min, RT), and the fluorescence signals were measured on a BioPlex^®^ 200 system (Bio-Rad Laboratories GmbH, Germany) using the following settings: Bead type: MagPlex^®^ beads; beads: 100 beads/region; sample timeout: 60 s; sample volume: 80 μL; gate settings: 7,500–15,000 (BioPlex^®^ Manager™ 5.0, Bio-Rad Laboratories GmbH, Germany). Signal detection on the BioPlex^®^ 200 System included determination of the autofluorescence of the beads and the fluorescence intensity of the bound detection antibodies. This allowed the differentiation of the individual protein extracts as well as the quantification of specific antibody levels.

#### Data analysis and statistical analysis

The xMAPr analysis tool (xMAPr 1.1.2; S. Michalik; https://michalik.shinyapps.io/xMAPr_app/ [30]) was used to derive a relative antibody concentration from the multiple data points of the serum titrations. Based on a non-linear regression model, the relative antibody concentration was calculated using the signal intensity of at least 100 beads (median fluorescence intensity, MFI) and the dilution factor according to the following formula:

$$\mathrm{Relative \, antibody \, concentration \, }\left[{\text{AU}}\right]= \, \frac{1}{2}{\text{MFI}}_{\text{max}} \, \, {\text{dilution factor}}_{\frac{1}{{2}}{{\text{MFI}}}_{\text{max}}}$$

Antibody profiling was performed with two batches of antigen-coupled beads. Batch correction was performed for each ECP individually using the human reference serum that was run on each plate. The relative antibody concentrations for batch 1 were multiplied with a batch correction factor (ratio of the median relative antibody concentration for the second batch divided by the median relative antibody concentration for the first batch). Missing value imputation based on a global LOESS fit over a single dilution was performed for missing relative antibody concentrations if curve fitting failed (1:2,500 dilution). A plate-to-plate normalization of the batch-adjusted and imputed relative antibody concentrations was performed using the human reference serum.

Since most of the PJI patients included in this study were admitted non-electively for urgent treatment of acute PJI symptoms, we could not obtain a pre-infection sample to determine patient-specific baseline antibody levels. We therefore used the serum samples obtained at the latest available time point, usually at the last clinical follow-up visit (f) after successful treatment as reference values for calculating ratios for the IA time course:

$$\mathrm{Ratio }\left(\frac{{\text{dx}}}{{\text{df}}}\right)= \left(\frac{\mathrm{relative \, antibody \, concentration \, at \, day \, x}}{\mathrm{relative \, antibody \, concentration \, at \, follow\text{--}\text{up}}}\right)$$

Statistical testing and data visualization were performed with GraphPad Prism (v8.0.1) and in R (v4.2.0; https://www.R-project.org/) in combination with the tidyverse package (v2.0.0) [33] and the patchwork package (v1.1.2; https://CRAN.R-project.org/package=patchwork). A two-tailed Mann–Whitney test was employed for comparison of two datasets. Pathogen-specific cut-off values were calculated by combining data from all 24 PJI and Non-PJI patients included in the study, and removing outliers (N = 129) using the robust regression and outlier removal (ROUT) method (using a coefficient Q = 0.1%, to apply a stricter threshold for defining outliers) [34]. The remaining data were used to calculate the ratios indicated above (ratio (dx/df)), and the pathogen-specific cut-off value was set at mean of ratio (dx/df) + 5 × SD (Table 2).

## Results and discussion

### Patient demographics and outcome of PJI therapy

A total of 21 male and 3 female patients were enrolled in the study, with 13 patients presenting with PJI (PJI patients). The average age of PJI patients was 70.9 years, while that of Non-PJI patients was 70.5 years (Table 1).

The clinical outcome of the PJI group showed that two-stage revision arthroplasty including eradication of PJI was relapse-free in 9 of 13 cases (69%) (Table 3). Two patients declined to undergo reimplantation surgery for personal reasons (PJI_04, PJI_09). One patient had continuously elevated infection parameters (serum CRP) and did not undergo reimplantation surgery for that reason (PJI_05). Moreover, one patient suffering from chronic cardiac failure died from a major adverse cardiovascular event between the first- and second-stage surgeries (PJI_10).

**Table 3:** Bacterial species detected by microbiological culture and IA, as well as clinical outcome for PJI patients (n = 13)

Patient ID	Sex	Age	Arthroplasty type	PJI classification	Microbiological culture*		IA		Clinical Outcome**	Clinical follow-up (months)
					Result	Species	Result	Species		
PJI_01	f	81	THA	Late	+	*E. faecium*, *S. epidermidis*	+	*S. epidermidis*, *S. warneri*	Relapse-free	15
PJI_02	m	54	THA	Delayed	−	N/A	−	N/A	Relapse-free	5
PJI_03	m	60	THA	Early	+	*F. magna*, *S. epidermidis*	+	*C. difficile, E. coli,* *P. aeruginosa*	Relapse-free	14
PJI_04	m	83	THA	Late	−	N/A	−	N/A	Reimplantation declined by patient	8
PJI_05	m	67	THA	Early	+	*S. hominis*, *E. cloacae*	−	N/A	No reimplantation due to increased serum CRP	8
PJI_06	m	59	THA	Late	+	*S. aureus*, *S. epidermidis*	+	*S. warneri*	Relapse-free	7
PJI_07	m	63	TKA	Late	−	N/A	+	*S. mitis*	Relapse-free	16
PJI_08	m	66	TKA	Late	−	N/A	−	N/A	Relapse-free	10
PJI_09	m	82	THA	Late	+	*K. pneumoniae*	+	*S. epidermidis,* *S. gallolyticus*	Reimplantation declined by patient	6
PJI_10	m	84	THA	Late	+	*K. pneumoniae*	+	*S. epidermidis,* *S. hominis*	Death due to cardiac failure	6
PJI_11	m	85	THA	Late	−	N/A	+	*M. catarrhalis*	Relapse-free	4
PJI_12	m	73	THA	Late	+	*S. epidermidis*, *C. acnes*	+	*S. epidermidis,* *S. warneri*	Relapse-free	3
PJI_13	m	65	THA	Late	−	N/A	−	N/A	Relapse-free	7

* from hip or knee joint

** the outcome was defined as relapse-free according to the following criteria: healed wound, no subsequent surgical intervention and no occurrence of PJI-related mortality

Abbreviations: -, negative; + , positive; CRP, C-reactive protein; IA, Infection Array; N/A, not available; THA, total hip arthroplasty; TKA, total knee arthroplasty

Culture-based microbiology detected a pathogen in 7 of 13 PJI cases (Table 3, Table 4), resulting in 46% culture-negative PJI. All microbiological cultures of the Non-PJI group remained negative. In sum, and taking the clinical classification of PJI as a measure, conventional culture-based microbiology achieved sensitivity of 54%, a specificity of 100%, a positive predictive value of 100% and a negative predictive value of 65% for the detection of PJI.

**Table 4:** Characteristics of the microbiological findings for PJI patients (n = 13)

Characteristic	PJI patients (n = 13)
**Microbiological culture results**	
Culture-positive PJI	7
Polymicrobial PJI	5/7
Culture-negative PJI	6
**Isolated bacterial species**	
*Staphylococcus aureus*	2
*Staphylococcus epidermidis*	5
*Staphylococcus hominis*	1
*Staphylococcus haemolyticus*	1
*Enterococcus faecium*	4
*Enterococcus faecalis*	2
*Finegoldia magna*	1
*Enterobacter cloacae*	1
*Candida* spp.	4
*Streptococcus agalactiae*	1
*Streptococcus dysgalactiae*	1
*Klebsiella pneumoniae*	3
*Cutibacterium acnes*	1
*Escherichia coli*	1
*Proteus mirabilis*	1

The high rate of culture-negative infections in our PJI group (6 of 13 cases) might be explained by the fact that our center is a tertiary referral center for septic orthopaedic surgery, and hence many patients had had a failed attempt at PJI treatment prior to transferal to our center. Therefore, a large percentage of patients in this cohort had received antimicrobial therapy before they were admitted to our institution.

### Multiplex-antibody detection using the IA

**Fig. 4:**
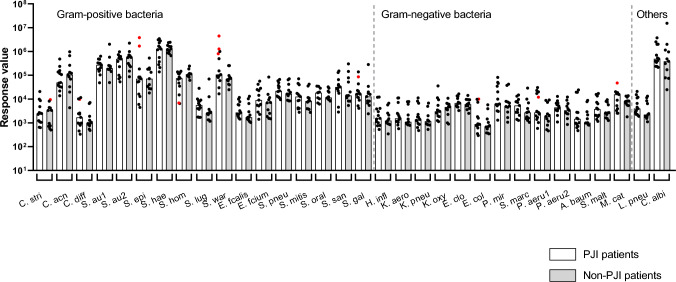
Pathogen-specific serum antibody levels in PJI patients and Non-PJI patients at the earliest available time point. Once the patients were enrolled in the study, a serum sample was obtained to measure pathogen-specific serum antibody levels using the 33-plex IA. Preoperatively, all PJI and Non-PJI patients showed basal levels of antibodies directed against the different pathogens, with no significant differences in pathogen-specific antibody levels between both groups. Specific antibody binding levels varied greatly between the tested pathogens. There was also pronounced variation in the patients’ responses to a given pathogen, reflecting their immune memory. Antibody levels that later declined during PJI treatment are highlighted in red (for explanation, please refer to Fig. 6). Abbreviations: A. baum, *Acinetobacter baumannii*; C. acn, *Cutibacterium acnes*; C. albi, *Candida albicans*; C. diff, *Clostridioides difficile*; C. stri, *Corynebacterium striatum*; E. clo, *Enterobacter cloacae*; E. col, *Escherichia coli*; E. fcalis, *Enterococcus faecalis*; E. fcium, *Enterococcus faecium*; H. infl, *Haemophilus influenzae*; K. aero, *Klebsiella aerogenes*; K. oxy, *Klebsiella oxytoca*; K. pneu, *Klebsiella pneumoniae*; L. pneu, *Legionella pneumophila*; M. cat, *Moraxella catarrhalis*; P. aeru1/ P. aeru2, *Pseudomonas aeruginosa*; P. mir, *Proteus mirabilis*; S. au1/ S. au2, *Staphylococcus aureus*; S. epi, *Staphylococcus epidermidis*; S. gal, *Streptococcus gallolyticus*; S. hae, *Staphylococcus haemolyticus*; S. hom, *Staphylococcus hominis*; S. lug, *Staphylococcus lugdunsensis*; S. malt, *Stenotrophomonas maltophilia*; S. marc, *Serratia marcescens*; S. mitis, *Streptococcus mitis*; S. oral, *Streptococcus oralis*; S. pneu, *Streptococcus pneumoniae*; S. san, *Streptococcus sanguinis*; S. war, *Staphylococcus warneri*

Using the IA, we were able to monitor the patients’ humoral immune response to all 32 PJI-pathogens included in the 33-plex. Preoperatively, at enrollment, all PJI and Non-PJI patients had measurable serum IgG binding against the 32 pathogens (Fig. 4), indicating immune memory. Their antibody binding profiles reflected their history of immunologic exposure to the pathogens, most of which are common members of the endogenous microbiota of the skin, oral cavity, respiratory or gastrointestinal tracts.

Interestingly, the average antibody binding intensity varied strongly between pathogens, the range exceeding 5 orders of magnitude between *Escherichia coli* (E. col) and *Candida albicans* (C. albi). The antibody responses to a given pathogen also varied widely between patients, with differences between minimum and maximum antibody levels ranging from a factor of 7 for *Streptococcus oralis* (S. oral) to a factor of 2248 for *Staphylococcus epidermidis* (S. epi). In 10/32 pathogens tested, the range of binding exceeded a factor of 100 (Fig. 4).

Preoperatively, we observed no significant differences in pathogen-specific IgG levels between the PJI and Non-PJI groups. Given the pronounced variability in the preoperative antibody binding patterns and assuming that the PJI patients were infected with different pathogens, this comes as no surprise. A patient’s immune system will acutely engage only the invasive pathogen(s), while antibody binding to all other pathogens will be similar in PJI and Non-PJI patients.

Using a similar approach in a prospective study of sepsis patients, we measured basal IgG binding to common sepsis pathogens prior to infection. The variability was comparable to that seen in PJI at enrollment. Infection then induced a marked and specific increase in IgG binding to the sepsis pathogen(s) over the course of 16 days [35].

### Patients showed individual antibody binding patterns in the IA

We then monitored the specific antibody levels during the course of PJI treatment up to 16 months after explantation surgery. In contrast to the sepsis patients, who were enrolled before infection [35], we usually did not observe pathogen-specific increases in serum IgG binding during the treatment of PJI. Since the PJI patients were already infected at enrollment, mostly with chronic infections, any infection-induced increases had probably occurred in the past, and infection-related antibody levels were at a high plateau at enrollment. One of the 13 PJI patients experienced a transient elevation in IgG binding to *Staphylococcus epidermidis* from day 75, 18 days after reimplantation (PJI_09; Suppl. Fig. S1), hinting at a secondary infection. Indeed, patient PJI_09 underwent a revision at day 68, a second explantation surgery at day 86, and then refused a second reimplantation.

**Fig. 5:**
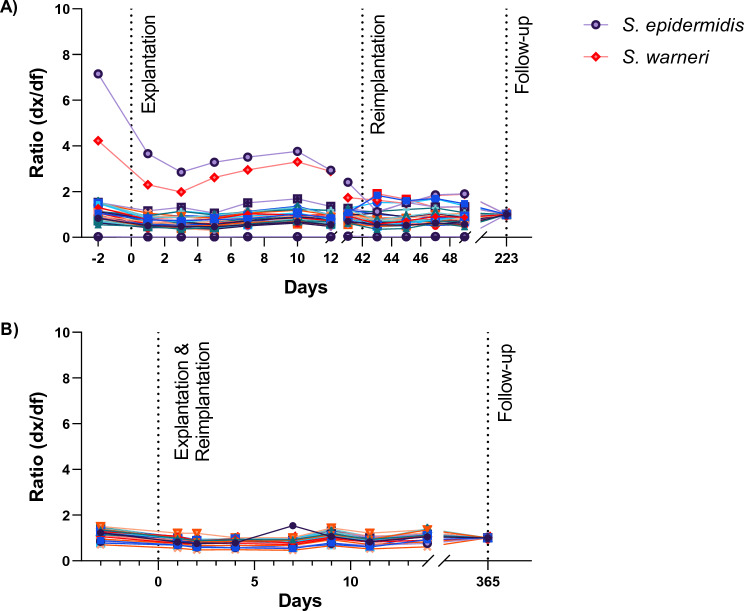
Kinetics of pathogen-specific antibody levels in PJI patients and Non-PJI controls. Relative pathogen-specific antibody concentrations were determined at the different time points (dx), and expressed as a ratio in relation to the follow-up sample (ratio (dx/df)). The time points of explantation, reimplantation and final follow-up visit are indicated (vertical lines). A pathogen-specific cut-off value was determined, and those pathogens, for which the ratio (dx/df) exceeded the cut-off value are highlighted in the figure legend. **A** Antibody binding kinetics in PJI patient PJI_01. The antibody levels for most bacteria remained stable throughout the study period, while antibody levels against *S. epidermidis* and *S. warneri* clearly declined over the study period, implicating both pathogens in the infection process. **B** Kinetics of pathogen-specific antibody concentrations in a Non-PJI patient (Non-PJI_01). Antibody levels against all pathogens remained stable over a year.

In contrast, the 33-plex IA revealed that antibody binding to some pathogens declined over the treatment period. This was observed in 8 of the 13 PJI cases, including 6 of the 9 PJI patients in whom two-stage septic arthroplasty revision surgery was successful (Fig. 5A; Suppl. Fig. S1), and in 1 of the 11 Non-PJI cases (Fig. S2). In the PJI patients the decrease in antibody binding was selective for 1–3 pathogens, prominently coagulase-negative staphylococci and *Streptococcus* spp., while antibody binding to all other pathogens remained stable (Fig. 5A, Suppl. Fig. S1). It is noteworthy that the antibody titers had strongly declined already at the time of reimplantation surgery.

In most Non-PJI patients (9 out of 11), antibody titers did not change during the whole observation period. Only in patient Non-PJI_07 the IgG binding to *Corynebacterium striatum* declined with similar kinetics as in the PJI patients, and patient Non-PJI_08 experienced a transient increase in IgG binding to *Haemophilus influenzae* (Fig. 5B, Suppl. Fig. S2).

We propose that a selective decrease in antibody binding to a pathogen signifies infection with this pathogen at patient enrollment and its resolution with successful treatment. This is in line with Bauer et al*.* who proposed multiplex-antibody detection for the post-therapeutic monitoring of PJI patients to complement the use of inflammatory markers [36]. They further suggest that such decline in pathogen-specific IgG binding may indicate the optimal time point for reimplantation [36]. Our data show that this approach can also monitor secondary bacterial invasion during treatment.

The French company Diaxonhit (Paris, France) has developed a commercial bead-based multiplex immunoassay (BJI InoPlex™) for pathogen diagnosis in PJI. It utilizes a selection of 16 recombinant antigens from three *Staphylococcus* species (*S. aureus*, *S. epidermidis*, and *S. lugdunensis*), *Streptococcus agalactiae* and *C. acnes* [37]. The assay showed sufficient to good diagnostic sensitivity in several clinical studies, but failed to provide an effective diagnosis of chronic PJI in complex microbiological situations [37–40].

### Comparison of microbiological and serological data

Comparison of the microbiological culture results with the immune signatures of infection (decline in antibody response), showed concordance in 6/13 PJI patients (46%) [2 × *S. epidermidis* (PJI_01, PJI_12); 4 × negative concordance (PJI_02, PJI_04, PJI_08, PJI_13)] (Table 3). In the remaining 7 PJI patients, microbiology and IA data were as follows: (i) both methods identified different causative pathogens (n = 4; PJI_03, PJI_06, PJI_09, PJI_10); (ii) the IA pinpointed one pathogen, while microbiological culture remained negative (n = 2; PJI_07, PJI_11); and (iii) microbiological culture implicated 2 pathogens, while IA data showed no changes in the antibody responses over time (n = 1; PJI_05).

In the Non-PJI group microbiological culture results and IA were concordantly negative in 9 out of 11 cases (82%; Table 3). In two Non-PJI patients, the IA detected a pathogen-specific antibody response, while the microbiological samples remained negative [1 × *Corynebacterium striatum* (Non-PJI_07); 1 × *Haemophilus influenzae* (Non-PJI_08)]. These differences underline that the pathogen-directed microbial culture and the host-directed IA measure complementary aspects of the infection: viable bacteria in the joint and an immune response to microbial invasion, respectively.

**Fig. 6:**
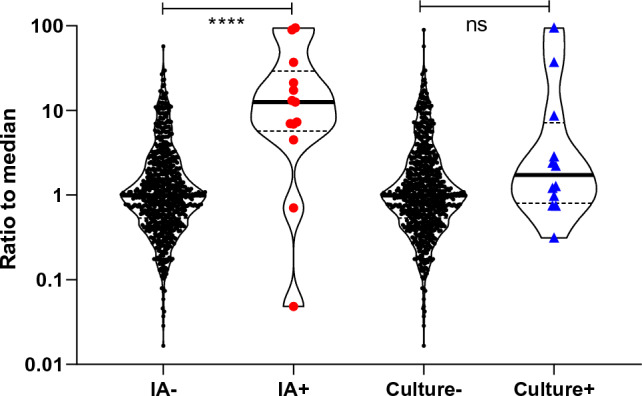
Correlation of basal antibody binding with antibody kinetics and microbiological culture results. Patients in whom antibody binding to a pathogen declined during PJI therapy (IA+) had exceptionally high antibody levels against this pathogen at baseline. These differed significantly from the basal levels of pathogen-specific antibodies which remained stable during PJI treatment (IA-). In contrast, baseline antibody binding to infectious agents did not differ significantly between pathogens with negative (Culture-) and positive (Culture +) microbiological culture. ****, p < 0.0001; ns, non-significant

Finally, we revisited the basal antibody levels before explantation surgery and compared them with the IA immune signatures of infection. Patients in whom antibody binding to a particular pathogen declined during therapy had exceptionally high antibody levels against this pathogen at baseline (Fig. 6, red dots). These were tenfold higher than the basal levels of the other antibody specificities, which remained stable during the course of clinical PJI treatment. In contrast, basal antibody binding to the pathogens identified by microbiological culture did not differ significantly from that to the other pathogens (Fig. 6, blue triangles). This strongly supports the idea that the antibody signature reflects an immune response to microbial invasion. It further suggests that very high specific antibody titers in the IA should raise suspicion of PJI.

Our pilot study shows that the multiplex IA is suitable for monitoring a patient’s specific antibody response to PJI and PJI treatment. The immunological approach has potential advantages over conventional methods for PJI diagnosis: The multiplex IA (i) is minimally invasive and therefore suitable for monitoring; (ii) provides rapid results; (iii) is less prone to sampling errors (false positive/negative results); (iv) differentiates between microbial contamination and invasion; (v) covers a wide spectrum of microbial strains that can be easily expanded; (vi) is sensitive because it tests antibody binding to all extracellular proteins of a pathogen; and (vii) is robust against antimicrobial therapy because it measures the host response to PJI. In the future, immunoproteomic analysis of the patients’ antibody responses may complement the standard diagnostic work-up to rule out PJI and monitor the treatment success in affected patients.

### Limitations

Larger patient numbers are required to assess the potential of IA for improving the monitoring of PJI treatment. With the data obtained from larger study cohorts, it will also be possible to determine the optimal sampling schedule required for a more efficient implementation of the IA. Another limitation of the IA is a possible cross-reactivity between closely-related pathogens, for instance within the genus *Staphylococcus*. Indeed, this might account for the discrepancy in patient PJI_06, where microbiology and IA identified different staphylococcal species. Moreover, the IA was able to identify only 8/13 PJI cases, which might indicate a low sensitivity of the IA for the detection of PJI. Furthermore, it has to be kept in mind that the IA measures a systemic immune response to a pathogen but does not indicate the site of invasion. However, it is not the authors’ intention to promote the IA as a PJI diagnostic tool. Our study provides the proof of principle that the IA can be used to monitor and gain insights into the individual patient’s dynamic humoral immune response to PJI.

## Conclusions

To our knowledge, this is the first study providing insights into the pathogen-specific immune response of PJI patients undergoing two stage septic hip or knee arthroplasty exchange. Based on the results of this pilot study, we conclude that the IA can be considered as a novel and valuable tool to complement conventional microbiological culture methods. It may serve to monitor the efficacy of PJI treatment, and provide valuable information for the differential diagnosis of PJI versus non-infectious causes of implant failure. In the future, it may help to predict the success or failure of PJI treatment to achieve optimal patient follow-up and help guide clinicians to timely interventions and adaptation of the treatment strategy.

## Supplementary Information

Below is the link to the electronic supplementary material.

Supplementary file1 (DOCX 1849 KB)

## Data Availability

The raw data supporting the conclusions of this article will be made available upon request by the authors, without undue reservation.
