# Supplementary material for: Investigation of the pathogen-specific antibody response in periprosthetic joint infection
Source: Infection. 2024 May 31;52(6):2325–37. doi: 10.1007/s15010-024-02285-y (PMC11621160; doi:10.1007/s15010-024-02285-y)
Supplement: Supplementary file 1 — Supplementary file1 (DOCX 1849 KB) [file 15010_2024_2285_MOESM1_ESM.docx]

Infection

Investigation of the pathogen-specific antibody response in periprosthetic joint infection

Viktor Janz^1,2^, Anastasia Rakow^1^, Leonie Schröder^3^, André Hofer^1^, Sergej Wiebe^1^, Janosch Schoon^1^, Stefan Weiss^4^, Barbara M. Bröker^3^, Georgi I. Wassilew^1^, Dina Raafat^3,5^

^1^Center for Orthopaedics, Trauma Surgery and Rehabilitation Medicine, University Medicine Greifswald, 17475 Greifswald, Germany

^2^Sporthopaedicum, 93053 Regensburg, Germany

^3^Institute of Immunology, University Medicine Greifswald, 17475 Greifswald, Germany

^4^Interfaculty Institute for Genetics and Functional Genomics, University Medicine Greifswald, 17475 Greifswald, Germany

^5^Department of Microbiology and Immunology, Faculty of Pharmacy, Alexandria University, 21521 Alexandria, Egypt

***Corresponding Author:**

Dr. Dina Raafat

Institute of Immunology, University Medicine Greifswald

F.-Sauerbruchstraße, 17475 Greifswald

e-mail: [dina.raafat@med.uni-greifswald.de](mailto:dina.raafat@med.uni-greifswald.de)

phone: (+49)-3834-865518; fax: (+49)-03834-865545

# Supplementary Information

# Supplementary Tables

Suppl. Table S1: Patient characteristics: overview of PJI and Non-PJI patients recruited for the study

| **Patient ID** | **Sex** | **Age at recruitment [years]** | **Arthroplasty type** | **Number of serum samples collected*** |
| --- | --- | --- | --- | --- |
| **A) PJI-group** | | | | |
| **PJI_01** | f | 81 | THA | 13 |
| **PJI_02** | m | 54 | THA | 17 |
| **PJI _03** | m | 60 | THA | 26 |
| **PJI _04** | m | 83 | THA | 7 |
| **PJI _05** | m | 67 | THA | 8 |
| **PJI _06** | m | 59 | THA | 22 |
| **PJI _07** | m | 63 | TKA | 20 |
| **PJI _08** | m | 66 | TKA | 17 |
| **PJI _09** | m | 82 | THA | 12 |
| **PJI _10** | m | 84 | THA | 4 |
| **PJI _11** | m | 85 | THA | 22 |
| **PJI _12** | m | 73 | THA | 19 |
| **PJI _13** | m | 65 | THA | 14 |
| **Total** |  |  |  | **201** |
| **B) Non-PJI group** | | | | |
| **Non-PJI_01** | f | 82 | THA | 9 |
| **Non-PJI _02** | m | 68 | TKA | 5 |
| **Non-PJI _03** | f | 59 | THA | 5 |
| **Non-PJI _04** | f | 50 | TKA | 6 |
| **Non-PJI _05** | m | 64 | TKA | 6 |
| **Non-PJI _06** | f | 83 | THA | 7 |
| **Non-PJI _07** | f | 78 | THA | 8 |
| **Non-PJI _08** | f | 79 | THA | 5 |
| **Non-PJI _09** | f | 65 | THA | 5 |
| **Non-PJI _10** | f | 64 | THA | 5 |
| **Non-PJI _11** | f | 84 | THA | 5 |
| **Total** |  |  |  | **66** |

* A total of 267 serum samples were analyzed using the IA.

Abbreviations: f, female; IA, Infection Array; m, male; PJI, periprosthetic joint infection, THA, total hip arthroplasty; TKA, total knee arthroplasty.

Suppl. Table S2: Extracellular proteins included in the 33-plex Infection Array (IA)-panel

| Nr. | Species / Protein | Strain^b^ | Abbreviation | Culture conditions | |
| --- | --- | --- | --- | --- | --- |
|  |  |  |  | Culture medium^c^ | Growth conditions |
|  | 1. **Gram-positive bacteria** | | | | |
| 1 | *Corynebacterium striatum* | DSM 20668 | C. stri | TSB | 37°C |
| 2 | *Cutibacterium acnes* | DSM 1897 | C. acn | TGB | anaerobic; 37°C |
| 3 | *Clostridioides difficile* | DSM 27147 | C. diff | BHI | anaerobic; 37°C |
| 4 | *Staphylococcus aureus* | USA300*ΔspaΔhla* | S. au1 | TSB+ | 37°C |
| 5 | *Staphylococcus aureus* | NCTC8325*Δspa* | S. au2 | TSB+ | 37°C |
| 6 | *Staphylococcus epidermidis* | RP62A | S. epi | TSB+ | 37°C |
| 7 | *Staphylococcus haemolyticus* | DSM 20263 | S. hae | TSB+ | 37°C |
| 8 | *Staphylococcus hominis* | DSM 20328 | S. hom | TSB+ | 37°C |
| 9 | *Staphylococcus lugdunsensis* | SL0902 | S. lug | TSB+ | 37°C |
| 10 | *Staphylococcus warneri* | DSM 20316 | S. war | TSB+ | 37°C |
| 11 | *Enterococcus faecalis* | ATCC 29212 | E. fcalis | TSB | 37°C |
| 12 | *Enterococcus faecium* | ATCC 51559 | E. fcium | TSB | 37°C |
| 13 | *Streptococcus pneumoniae* | TIGR4Δcps | S. pneu | THY | no shaking; 37°C; 5% CO_2_ |
| 14 | *Streptococcus mitis* | DSM 12643 | S. mitis | TSB | no shaking; 37°C; 5% CO_2_ |
| 15 | *Streptococcus oralis* | DSM 20627 | S. oral | TSB | no shaking; 37°C; 5% CO_2_ |
| 16 | *Streptococcus sanguinis* | DSS-10 | S. san | TSB | no shaking; 37°C; 5% CO_2_ |
| 17 | *Streptococcus gallolyticus* | CCUG 35224T | S. gal | TSB | no shaking; 37°C; 5% CO_2_ |
|  | 1. **Gram-negative bacteria** | | | | |
| 18 | *Haemophilus influenzae* | DSM 24049 | H. infl | HTM | 37°C, 5% CO_2_ |
| 19 | *Klebsiella aerogenes* | DSM 30053 | K. aero | TSB | 37°C |
| 20 | *Klebsiella pneumoniae* | ATCC 700721 | K. pneu | TSB | 37°C |
| 21 | *Klebsiella oxytoca* | DSM 5175 | K. oxy | TSB | 37°C |
| 22 | *Enterobacter cloacae* | DSM 30054 | E. clo | TSB | 37°C |
| 23 | *Escherichia coli* | ATCC 47076 | E. col | TSB | 37°C |
| 24 | *Proteus mirabilis* | DSM 4479 | P. mir | TSB | 37°C |
| 25 | *Serratia marcescens* | DSM 30121 | S. marc | TSB | 37°C |
| 26 | *Pseudomonas aeruginosa* | PAO1 | P. aeru1 | TSB | 37°C |
| 27 | *Pseudomonas aeruginosa* | PA6077 | P. aeru2 | TSB | 37°C |
| 28 | *Acinetobacter baumannii* | DSM 105126 | A. baum | TSB | 37°C, 1-2 days |
| 29 | *Stenotrophomonas maltophilia* | SM1404 | S. malt | TSB | 28°C, 1-2 days |
| 30 | *Moraxella catarrhalis* | DSM 9143 | M. cat | BHI | 37°C; 5% CO_2_ |
|  | 1. **Atypical bacteria** | | | | |
| 31 | *Legionella pneumophila* | DSM 25061 | L. pneu | BYE | 37°C; 5% CO_2_; 2 days |
|  | 1. **Fungi** | | | | |
| 32 | *Candida albicans* | SC5314 | C. albi | SDB | shaking; 25°C |
| 33 | Tetanus Toxoid^a^ | - | TT | - | - |

^a^ Tetanus Toxoid (TT; Statens Serum Institute, Denmark) was included as a control antigen.

^b^ ATCC, American Type Culture Collection; DSM, Deutsche Sammlung von Mikroorganismen und Zellkulturen (German Collection of Microorganisms and Cell Cultures GmbH; www.dsmz.de)

^c^ BHI, Brain Heart Infusion broth; BYE-Medium, Buffered Yeast Extract; HTM, Haemophilus Test Medium; SDB, Sabouraud Dextrose Broth; TGB, Thioglycollate Medium enriched with vitamin K1 and hemin; THY, Todd-Hewitt broth supplemented with 0.5% yeast extract; TSB, Tryptic Soy Broth; TSB+, TSB+600 µM 2,2‘-Bipyridyl.

Supplementary Figures

Suppl. Fig. S1: Kinetics of pathogen-specific antibody production in PJI patients (PJI_02 – PJI_13). Relative pathogen-specific antibody concentrations were determined at the different time points (dx), and expressed as a ratio in relation to the follow-up sample, which was set at 1 (ratio (dx/df)). The time points of explantation, reimplantation and clinical follow-up are depicted in the figure (vertical lines). A pathogen-specific cut-off value was determined, and those pathogens, for which the ratio (dx/df) exceeded the cut-off value are highlighted in the respective figure legend.

Suppl. Fig. S2: Kinetics of pathogen-specific antibody production in Non-PJI patients (Non-PJI_02 – Non-PJI_11). Relative pathogen-specific antibody concentrations were determined at the different time points (dx), and expressed as a ratio in relation to the follow-up sample, which was set at 1 (ratio (dx/df)). The time points of explantation/reimplantation and clinical follow-up are depicted in the figure (vertical lines). A pathogen-specific cut-off value was determined, and those pathogens, for which the ratio (dx/df) exceeded the cut-off value are highlighted in the respective figure legend. For most of the Non-PJI patients (9/11), relative antibody concentrations for all pathogens remained stable for the study period (41-365 days).
